# SARS-CoV-2, Early Entry Events

**DOI:** 10.1155/2020/9238696

**Published:** 2020-11-24

**Authors:** James P. Chambers, Jieh Yu, James J. Valdes, Bernard P. Arulanandam

**Affiliations:** ^1^South Texas Center for Emerging Infectious Diseases, The University of Texas at San Antonio, San Antonio, TX, USA; ^2^MSI STEM Research and Development Consortium, Washington, DC, USA

## Abstract

Viruses are obligate intracellular parasites, and host cell entry is the first step in the viral life cycle. The SARS-CoV-2 (COVID-19) entry process into susceptible host tissue cells is complex requiring (1) attachment of the virus via the conserved spike (S) protein receptor-binding motif (RBM) to the host cell angiotensin-converting-enzyme 2 (ACE2) receptor, (2) S protein proteolytic processing, and (3) membrane fusion. Spike protein processing occurs at two cleavage sites, i.e., S_1_/S_2_ and S_2_′. Cleavage at the S_1_/S_2_ and S_2_′ sites ultimately gives rise to generation of competent fusion elements important in the merging of the host cell and viral membranes. Following cleavage, shedding of the S_1_ crown results in significant conformational changes and fusion peptide repositioning for target membrane insertion and fusion. Identification of specific protease involvement has been difficult due to the many cell types used and studied. However, it appears that S protein proteolytic cleavage is dependent on (1) furin and (2) serine protease transmembrane protease serine 2 proteases acting in tandem. Although at present not clear, increased SARS-CoV-2 S receptor-binding motif binding affinity and replication efficiency may in part account for observed differences in infectivity. Cleavage of the ACE2 receptor appears to be yet another layer of complexity in addition to forfeiture and/or alteration of ACE2 function which plays an important role in cardiovascular and immune function.

## 1. Introduction

Coronaviruses (CoVs) are enveloped, single-stranded RNA viruses that cause gastrointestinal, respiratory, and neurological symptoms in several mammalian species and birds [1]. Prior to the outbreak of the severe acute respiratory syndrome coronavirus (SARS-CoV) in Southern China in 2002-2003 which caused a fatal pneumonia in approximately 10% of those infected, CoVs in large part were considered harmless in humans [2, 3]. However, due to emergence of the highly pathogenic MERS-CoV (Middle East Respiratory Syndrome Coronavirus) in 2012 [4] and the SARS-CoV recently discovered in Wuhan, China (SARS-CoV-2/COVID-19 [5]), the causative agent of an atypical fulminant pneumonia now referred to as the worst pandemic disease of modern times [6], this view has changed.

Viruses are obligate intracellular parasites. As such, host cell entry is the first step in the viral life cycle. The CoV genome encodes three surface proteins, i.e., spike (S), membrane (M), and envelope (E) [7]. However, it is the S protein that is intimately involved in viral entry initially binding to the host cell surface receptor prior to fusion of viral and host cell membranes. CoVs use a broad range of receptors for entry into target cells which is summarized in Table 1. Receptor recognition is considered the key initial event that determines viral infectivity, pathogenesis, and host range.

Much insight into the CoV entry process was gleaned from comparison of compounds investigated for their ability to inhibit SARS-CoV entry [14]. Cathepsin L, an endosomal cysteine protease, was shown to be required for initial proteolytic cleavage of SARS-CoV S protein [15, 16]. Initially, it was theorized that binding of SARS-CoV S protein to the host cell receptor triggers subtle conformational changes within the S protein. In turn, these changes render the S protein susceptible to host cell-mediated proteolytic cleavage, liberation of a fusion peptide, and subsequent merging of viral and host cell membranes, i.e., host cell entry [7]. These early events which have been elucidated to varying degree are the focus of this review.

## 2. SARS-CoV-2 Binds to Host Cell ACE2 Receptor via the Spike (S) Protein

The initial step in the SARS-CoV-2 entry cascade is S protein binding to the host cell surface receptor, i.e., angiotensin-converting enzyme 2 (ACE2) [12]. Using Hela cells that expressed ACE2 proteins, Zhou and coworkers concluded from infectivity studies that SARS-CoV-2 utilized ACE2 for cell entry, and cells that did not express ACE2 receptor did not facilitate viral entry into the host cell thus supporting ACE2 as being the receptor through which SARS-CoV-2 gains entry [17]. Similarly, Walls, Letko, and Wan demonstrated ACE2 mediated SARS-CoV-2 entry into cells [18–20]. Consistent with the fact that both SARS-CoV and -CoV-2 S proteins contain highly conserved amino acid residues essential for ACE2 receptor binding [21], most of these residues are absent from the S protein of SARSr-CoV (bats) previously observed not to use ACE2 for entry [22–24].

## 3. The CoV Spike (S) Protein Undergoes “Priming” and “Activation” Cleavages

**Figure 1:**
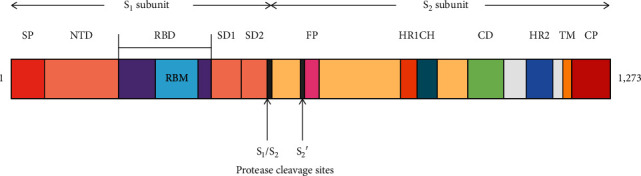
Schematic of the CoV S protein primary structure and cleavage sites. Arrows indicate cleavage sites (S_1_/S_2_) resulting in S_1_ and S_2_ subunits with a cleavage site (S_2_′) located in the S_2_ subunit. FP = fusion peptide; HR1 and HR2 = heptad repeat regions 1 and 2; TM = transmembrane anchor; CP = cytoplasmic domain; NTD = N-terminal domain; RBD = receptor-binding domain; RBM = receptor-binding motif; SP = signal peptide. Taken from Depfenhart, M., de Villiers, D., Lemperle, G., et al., Inter. Emerg. Med., vol. 15, pp. 801–812, 2020.

The CoV S protein is a Class I viral fusion protein synthesized as a single-chain precursor of approximately 1,300 amino acids [25]. It is the structural component giving the “crown-like” appearance from which the original name “coronavirus” was derived. The functional organization of the CoV S protein closely resembles that of other viral entry proteins, e.g., the influenza hemagglutinin (HA) and human immunodeficiency virus envelope protein [26]. Class I fusion proteins form homotrimers, and each monomer consists of two domains, S_1_ and S_2_. The S_1_ domain facilitates binding to the target cell receptor. The S_2_ domain is responsible for host cell-viral membrane merging [27]. The S protein is extensively glycosylated and shown important for proper protein folding, modulation of host cell protease substrate accessibility, and antibody binding [28–32]. Shown in Figure 1 is a schematic of the S protein primary structure and prerequisite proteolytic cleavage into S_1_ and S_2_ subunits.

The first cleavage (S_1_/S_2_ site) referred to as “priming” gives rise to S_1_ (surface) and S_2_ (transmembrane) functional subunits which remain noncovalently bound in a “prefusion” conformation [25]. Regardless of the cellular site, the protein, or the proteases involved, “priming” and “activation” (S_2_′ site cleavage) convert the fusion protein to a fusion-competent state [33, 34]. The second cleavage, i.e., activation, results in activation of membrane fusion. An early consequence of “activation” is the repositioning of the viral fusion peptide (or loop) that engages the target membrane lipid bilayer. The fusion peptide which is initially buried and inaccessible becomes exposed and repositioned, i.e., inserted into the target membrane forming the defining “prehairpin” fusion intermediate conformation.

### 3.1. S_1_/S_2_ Priming Cleavage

CoV S proteins are unusual in that they have multiple cleavage sites and multiple motifs. Thus, they function as a substrate for a wide variety of host cell proteases [35] comprising different families, e.g., the cathepsins [36], trypsin-like serine proteases such as members of the transmembrane serine protease (TTSP) family [37–40], and the furin-like proprotein convertases [35, 41] which give CoVs broad flexibility to invade new cell types, tissues, and host species.

Initial SARS-CoV-2 proteolytic processing in human cells has been associated with recognition of a polybasic (several arginine residues, i.e., -RRAR_685↓_-) furin site at the S_1_/S_2_ cleavage site [42] implicating involvement of multiple proteases affecting viral infectivity and host range. The polybasic furin site is absent in SARS-CoV and instead contains a single Arg residue [39, 43–45]. A similar polybasic furin site has been found in HA proteins of highly virulent avian and human influenza viruses [46]. Based on inhibition by camostat mesylate in lung cells, SARS-CoV-2 S_1_/S_2_ “priming” cleavage between the RBD and fusion peptide has been shown to be dependent on the human serine protease TMPRSS-2 (Transmembrane Protease Serine 2) [47], a trypsin-like protease that has been shown to cleave monobasic, i.e., R↓ sites.

The S_1_/S_2_ cleavage site exhibits different motifs among CoVs with many of them displaying cleavage specificity after a basic residue [42] ensuring that “priming” is carried out by different host cell proteases. Because SARS-CoV S protein can be cleaved by TMPRSS-2 which exhibits trypsin-like specificity, it is clear that trypsin can serve as a substitute protease. In either case, arginine 667 is critical for initial SARS-CoV S protein “priming” by TMPRSS-2 although this residue appears to be dispensable for TMPRSS-2 “activation” [48]. Conversely, arginine 797 is required for SARS-CoV “activation” by trypsin [49]. However, it is clear that arginine 667 and arginine 797 are required for SARS-CoV S protein cleavage by TMPRSS-2 [48]. With different S_1_/S_2_ cleavage site motifs and specificities, the assignment of a protease to a specific event, e.g., “priming” versus “activation,” is often difficult. As indicated above, TMPRSS-2 has been implicated in the “priming” process; however, the recent report of Bestle and coworkers clearly indicates S_2_′ cleavage, i.e., “activation” of SARS-CoV 2 in human airway cells occurs via TMPRSS-2 after “priming”, i.e., furin cleavage at the S_1_/S_2_ priming site [49].

Interestingly, while some SARS-CoV data does not support furin playing a role in S protein cleavage activation, insertion of a furin cleavage site at the S_1_/S_2_ junction has been shown to enhance cell-cell fusion without affecting viral entry [50]. A novel feature of the SARS-CoV-2 S protein setting it apart from SARS-CoV and SARSr-CoV (bats) is the “cleaving” of the furin cleavage site at the S_1_/S_2_ boundary during synthesis. However, recent data by Walls and coworkers indicate that cleavage during S protein biosynthesis is not necessary for S-mediated entry under the conditions examined and speculate that this may contribute to expanding the tropism of this virus [18]. Unique to the SARS-CoV-2 polybasic furin cleavage site is a leading proline that creates a turn [51]. Some have suggested this to be a possible O-type “mucin-like” glycosylation site (Ser673, Thr678, and Ser686), the presence and function of which remain to be established [52]. Bagdonaite and coworkers point out that such mucin-like domains flanking the cleavage site could possibly shield it and/or other key SARS-CoV-2 functional residues making them inaccessible [53]. Of related importance, efficient furin-like cleavage of MERS-CoV S protein has been shown to be important in MERS-like CoVs from bats and their infection of human cells [22].

Importantly, the first cleavage event in concert with host receptor “binding” promotes further cleavage at the S_2_′ site [44]. It appears that the engagement of specific CoV S protein proteases is dependent on protease availability, i.e., membrane location, and orientation. TMPRSS-2 CoV S protein activation appears to require specific spatial relationships between the viral S protein and membrane-bound protease(s). Glowacka [39] observed TMPRSS-2 activation only when the substrate, i.e., S protein, and protease are located in different membranes suggesting a trans-like cleavage. Conversely, it has been shown that TMPRSS-2 can cleave SARS-S when both proteins are localized in the same membrane, i.e., a cis-cleavage that has been hypothesized to result in shedding of soluble SARS-S protein into the extracellular space [54].

### 3.2. S_2_′ Activation Cleavage

Multiple proteases appear capable of participating in this process. In contrast to the S_1_/S_2_ site, a furin-like S_2_′ cleavage site (KR↓SF) downstream of the internal fusion peptide is identical in SARS-CoV and SARS-CoV-2 [55]. Coutard and coworkers have proposed that one or more furin-like enzymes cleave the S_2_′ site (KR↓SF) [42]. Protease specificity and location are important determinants as evidenced by S_2_′ proteolytic fusion activation occurring in several cellular compartments [56]. TMPRSS-2 processing of SARS-CoV S is principally at the cell membrane, whereas furin-mediated processing occurs at the cell surface and in the early endosome [35, 41, 56]. It appears that redundancy is built into the CoV S protein with both furin and related proprotein convertase recognition of polybasic cleavage sites [56].

**Figure 2:**
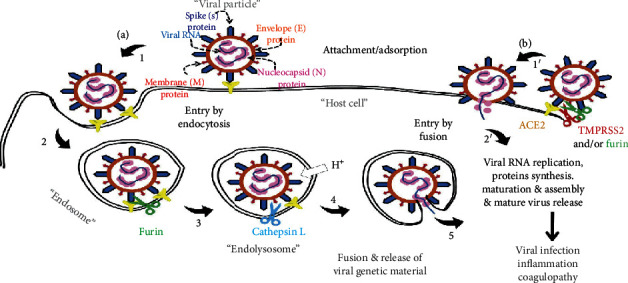
SARS-CoV-2 host cell entry. Entry begins with S protein binding to the host cell ACE2 receptor. (a) Endocytosis-mediated entry entails S protein priming/activation reactions occurring in the endosome/endolysosome. (b) Direct fusion entry is mediated by furin and/or TMPRSS-2 as well as other trypsin-like proteases. Taken from Al-Horani, R. A., Kar, S., and Aliter, K. F., Int. J. Mol. Sci., vol 21 (15), 5224, 2020.

There exist many variations of the canonical furin cleavage site in addition to the 20 amino acid stretch that surrounds the cleavage site which determines binding specificity [57]. Interestingly, bioinformatic analyses and functional studies have uncovered more than 100 furin cleavage sites in mammalian proteins [58]. While most furin targets are “activated” following cleavage, furin cleavage has also been shown to “inactivate” their respective targets, a possible means of viral entry proteolytic specificity control remaining to be explored [59, 60]. Depending on which virus strain and cell type are used for infection, CoV S “activation” by multiple proteinases including furin, trypsin, cathepsins, TMPRSS-2/4, and human airway trypsin-like protease (HAT) has been described [35, 37, 38, 47, 55, 61–63]. Shown in Table 2 and Figure 2 is an overview of proteolytic processing of SARS-CoV-2 S protein and viral entry.

## 4. The S_1_ Subunit Is Heavily Involved in Host Cell Receptor Binding

The CoV S_1_ subunit C terminus is comprised of a core of *β*-rich domains (designated A, B, C, D, and 0) and a globular external region in which reside amino acids important for receptor binding [12] as well as stabilization of the membrane-anchored S_2_ prefusion subunit that contains the fusion elements [64–69]. Although domain B exhibits the highest sequence variability, it contains the *β*-sheet core subdomain that mediates receptor-specific binding interactions [70–76].

The S_1_ subunit comprises the apex of the S trimer within which reside at the C terminus the conserved amino acid residues collectively referred to as the receptor-binding domain (RBD). The RBD contains the structural information required for cell surface ACE2 receptor binding [21, 43, 73, 76–78]. The receptor-binding motif (RBM) is the region (carboxy-terminal half of the RBD) that contains the residues that interface with the host ACE2 receptor [21]. For SARS-CoV-2, the S trimer exists in distinct conformational states arising from opening of the B domain at the trimer apex [18]. Trimer “opening” exposes the receptor recognition motifs/elements that are otherwise buried and required for host cell receptor engagement which leads to initiation of required fusion peptide conformational changes [29, 30, 66, 69, 79].

Located at the N-terminus of the S protein is the signal peptide required for introduction of nascent S protein into the host cells' secretory ER pathway [54] where it is extensively glycosylated [12]. Consistent with the theme that glycosylation may restrict protease accessibility, the region surrounding the S_1_/S_2_ and S_2_′ cleavage sites is less densely glycosylated [29, 30].

## 5. The S_2_ Subunit Is Heavily Involved in Membrane Fusion

The S_2_ subunit is comprised of *α*-helices, an antiparallel core *β*-sheet, a *β*-rich connector domain, and a stem helix leading to the heptad repeat 2 and transmembrane region [12]. More conserved than S_1_, S_2_ contains the S_2_′ “activation” proteolytic site located immediately upstream of the fusion peptide [44, 56]. The fusion peptide, a short segment of the larger fusion protein, has multiple cleavage sites and is comprised primarily of 15–25 hydrophobic amino acids. The “activation” cleavage event gives rise to the mature (having undergone extensive irreversible conformation changes [80, 81]) hydrophobic fusion peptide that is inserted into the host target cell membrane [35, 44, 54].

Consistent with interfacial hydrophobicity analyses and peptide library scanning data, residues 770–788 located immediately upstream of the S_2_′ cleavage site are thought to be the fusion peptide [27]. The sequence (residues 873–888) upstream of the heptad repeat region 1 (HR1) forms the “internal” fusion peptide [80] which is essential in mediating membrane merging during which the bilayer lipid principally becomes more ordered [81]. Fusion peptide insertion is considered overall to have a dehydration effect; whereby, removal of the repulsive force between the opposing membranes allows them to be proximally positioned and thus approach one another prior to actual fusion [82, 83]. Interestingly, the fusion peptides of SARS-CoV and SARS-CoV-2 are identical [42]. SARS-CoV S protein-induced membrane fusion has been shown to be calcium-dependent with higher calcium concentration enhancing the membrane ordering effect [81]. Calcium with its positive charge is thought to enhance membrane fusion by electrostatically interacting with lipid bilayer negatively charged head groups, thus decreasing the electrostatic repulsion of the two opposing membranes that are in close proximity prior to fusion. Following membrane fusion, Class I fusion proteins adopt a well-defined coiled structure referred to as a “6-helix bundle” or 6HB.

## 6. SARS-CoV Entry Is Either “Early” or “Late” Depending on Entry Pathway

As previously indicated, the proteolytic cleavage events essential for CoV entry appear confusing due in large part to the many different cell types studied. In typical cell culture systems, SARS-CoV gains entry via clathrin and nonclathrin pathways following receptor engagement [84, 85], but treatment of cells with trypsin and/or trypsin-like proteases (TTSPs) gives rise to entry by S protein-mediated fusion [25, 40, 86, 87] which in some cases has been shown to be cell type-dependent [48]. This pathway of entry in mouse lung epithelium (the site of SARS-CoV and -CoV-2 infection) is thought to be TTSP mediated as evidenced by the fact that serine protease inhibitors reduce SARS-CoV infection [48, 88].

An important feature of SARS-CoV entry in cultured cells is that it begins after a lag period [89] suggesting endosomal maturation is required. Although CoVs have been shown to be internalized via receptor-mediated clathrin-dependent, caveolin-dependent, or other pathways [41, 84, 90], SARS-CoV has been shown to enter via both clathrin-dependent and clathrin/caveolae-independent entry pathways [84]. Based on in vitro and in vivo studies, viral entry via cell surface receptor recognition and membrane fusion is referred to as “early” [27] (cf. Figure 2, SARS-CoV-2 host cell entry). Conversely, if entry occurs via the endosome requiring endosome maturation (cathepsin-driven), such entry is referred to as “late” [88]. In “late” entry, the virus is first endocytosed and subsequently cleaved by furin proprotein convertases [35, 45], cathepsin L [44, 45, 47, 61], and/or cathepsin B [51]. It is important to note that the protease-enriched endolysosomal environment can also generate inactivating CoV S protein cleavages resulting in decreased entry efficiency [88]. Due to lysosomal and plasma membranes having unique lipid component profiles, such differences can give rise to differential proteolytic effects [91]. Additionally, other factors may also come into play, for example, the formation of ternary complexes (receptor-tetraspanin-protease complex) as is the case for MERS-CoV cell entry [88].

## 7. The SARS-CoV S Protein Is Not the Only Protein Subjected to Proteolytic Cleavage

TMPRSS-2 and the metalloprotease (a disintegrin and metalloproteinase domain 17, ADAM17) have been shown to cleave the ACE2 receptor close to its transmembrane domain [7, 92, 93]. This cleavage supposedly results in ACE2 receptor shedding (not to be confused with removal of the S_1_ crown) and suggests that TTSPs and other proteases can impact S protein driven entry by ways other than S protein priming/activation [93, 94]. During the shedding process, the protease (referred to as Sheddase) cleaves the membrane protein substrate close to or within its transmembrane domain resulting in release of a soluble extracellular domain (ectodomain) from the membrane and a fragment that remains bound to the membrane [95].

## 8. Following Binding and Cleavage, Large Conformational Changes Occur

Following binding and cleavage, the CoV S protein exists in a metastable “prefusion” conformation that undergoes significant structural rearrangement for fusion of viral and the host cell membranes [25, 96]. Binding of the S_1_ subunit to the host cell receptor triggers this process. The S_1_ RBD undergoes hinge-like conformational movements that transiently hide or expose RBD determinants which facilitate engagement of the host cell receptor [97]. These movements or states are referred to as “up” and “down” conformations corresponding to receptor accessible (open) and receptor-inaccessible (closed), respectively [30, 33, 64, 67]. As the RBD undergoes this hinge-like conformational change, the S_1_ subunit appears to change shape, i.e., to “breathe”. This receptor-mediated triggering mechanism is thought to be conserved among Coronaviridae [30, 65]. Current data suggest the S protein trimers found in highly pathogenic human coronaviruses exist in partially opened states, whereas less or nonpathogenic human coronaviruses remain largely closed.

Kirchdoerfer and Walls postulated that cleaved S_1_ and S_2_ subunits interact [68, 70]. This was shown to be the case by stabilization of a “prefusion” S_2_ conformation. Proteolytic cleavage frees the fusion peptide allowing removal of the S_1_ crown and subsequent refolding of the fusion elements [69]. Interaction of the S_1_ B domain in the prefusion “closed” conformation with S_2_ results in stabilizing the “spring-loaded” metastable prefusion conformation. It appears that the connecting event between receptor engagement and S_1_/S_2_ and S_2_′ cleavage is removal of the S_1_ crown which brings about conformational changes in S_2_ and fusion peptide and subsequent insertion (approximately 100 Angstroms) into the target membrane [68, 69]. The refolding of S_2_ from the prefusion “spring loaded” to postfusion ground state conformation is thought to be the source of free energy bringing the viral and host membranes in close proximity for membrane merger [26].

## 9. SARS-CoV-2 Appears to Bind with Higher Affinity to the ACE2 Receptor

Compared to SARS-CoV, SARS-CoV-2 appears to be more easily transmitted from human to human [98–100]. Yet, the overall conformation of SARS-CoV and SARS-CoV 2 S protein RBD is similar with only minor differences observed in their respective “down” conformation [97]. Although they share the same functional host cell receptor, the binding affinity of SARS-CoV-2 for the ACE2 receptor was observed to be higher than that of SARS-CoV [19, 101]. This may in part account for the ease of infectivity by SARS-CoV-2 compared to SARS-CoV. Interestingly, Walls and coworkers using the S protein B domain from SARS-CoV and SARS-CoV-2 as binding ligand observed greater than a 4- and 4.4-fold decreased *K*_D_ and *k*_off_ value, respectively, for SARS-CoV-2 [18]. Although consistent with a longer “on” rate for SARS-CoV-2, this appears not to be the case since *k*_on_ values are almost identical for SARS-CoV-2 and SARS-CoV suggesting that the difference in infectivity is not as much as that of binding but perhaps one of efficiency of replication in the host cell.

## 10. Binding Is Not Everything

While viral S protein RBD-host cell receptor specificity is a well-established host range determinant, considerable data support the major role that host protease processing plays as a species barrier [22, 101, 102]. Following the initial 2012 SARS-CoV outbreak, emergence in humans was principally attributed to mutations within the RBD. Yet, there now exists a body of work indicating that circulating zoonotic SARS-like viruses in Southeast Asian bats are capable of infecting human cells by binding to ACE2 receptors without adaptation suggesting that CoV S protein receptor-binding specificity is not the only barrier to CoV emergence [102, 103]. Although the absence of receptor-binding or compatible host protease activity restricts infection with certain zoonotic strains, it now appears that such barriers can be overcome by participation of ubiquitous host cell proteases. Menarchery and coworkers point out that proteolytic cleavage of the MERS-CoV S protein may be the primary infection event [22] suggesting yet to be described intricate cleavage-binding connections.

## 11. Differences in SARS-CoV-2 S Protein RBD May Explain Differences in Infectivity

A key to understanding the difference between SARS-CoV and SARS-CoV-2 and resulting disease may reside in subtle structural differences in receptor recognition elements. The SARS-CoV-2 S protein RBD binding contacts for the ACE2 receptor are similar to those observed for SARS-CoV [20, 54]. Although the S protein RBD is the most variable part of the CoV genome [16, 103], six RBD amino acid residues have been shown to be critical for binding to the ACE2 receptor and, thus, determination of host range [20]. Comparing the respective RBD residues (Tyr442, Leu472, Asn479, Asp480, Thr487, and Tyr491) in SARS-CoV to corresponding residues (Leu455, Phe486, Gln493, Ser494, Asn501, and Tyr505) in SARS-CoV-2 indicates that 5 of 6 residues differ. While experimental data support increased SARS-CoV-2 binding affinity to the ACE2 receptor, computational assessment suggests the interaction is not ideal nor is the predicted sequence consistent with that shown for optimal SARS-CoV receptor binding [20].

Thus, there must be other considerations that account for the observed binding affinity. Using SARS-CoV and SARS-CoV-2 crystal structures, Shang and coworkers have observed changes in the S protein RBD receptor-binding ridge which consists of residues 482–485 (Gly-Val-Glu-Gly) [104]. Such changes allow the ridge to be more compact thus achieving better contact with the N-terminal helix of the ACE2 receptor by the SARS-CoV-2 RBD. Several amino acid residue changes are present in the SARS-CoV-2 RBD that stabilize the virus binding regions at the RBD/ACE2 interface. In SARS-CoV-2, insertion of the more hydrophobic Phe486 R-group into a hydrophobic RBD pocket forms a stronger contact than does the less hydrophobic leucine R-group of the corresponding RBD in SARS-CoV. Previously, it was shown that two lysine R-groups must be accommodated in a hydrophobic environment. Thus, lysine R-group charge neutralization is key to CoV binding to the ACE2 receptor [105, 106]. In SARS-CoV-2, this is achieved with Gln and Leu substitutions at positions 493 and 455, respectively, and Asn at position 501.

Further evidence supporting increased binding affinity of the SARS-CoV-2 RBD is derived from molecular modeling data indicating increased flexibility of a distinct loop with glycine replacing the rigid and restrictive proline residue R-group observed in SARS-CoV. [107]. Although a high degree of homology exists between SARS-CoV and SARS-CoV-2, monoclonal antibodies made against a recombinant SARS-CoV-2 RBD S protein fragment which was shown by interferometry to have folded correctly did not cross-react with the SARS-CoV RBD. This is consistent with the RBD variations observed by Wan and coworkers [20]. Subtle variations within the S_1_ RBD and the host cell receptor can dramatically impact cross-species transmission of coronaviruses. Two lysine residues (31 and 353) are critical for SARS-S protein binding to the human ACE2 receptor [106, 108]. Substitution at position 353 with histidine in the murine ACE2 receptor renders this protein unsuitable for efficient SARS-S protein binding [21]. Similarly, the rat ACE2 homologue contains a glycosylated asparagine residue at position 82 which sterically blocks SARS-CoV S protein host receptor interaction.

## 12. Conclusions

SARS-CoV-2 host cell entry is a complex process involving binding of the virus RBM to the ACE2 receptor, and proteolytic processing giving rise to large conformational changes in the S protein prior to required membrane fusion. Identification of specific protease involvement has been difficult due to the many cell types used and studied. It appears that viral entry via S protein attachment to the cell surface ACE2 receptor followed by membrane fusion is dependent on (1) furin and (2) TMPRSS-2 proteases acting in tandem, whereas entry by endocytosis requires multiple proteases, e.g., furins, cathepsins, and others. Although currently not clear, increased SARS-CoV-2 S receptor-binding affinity and replication efficiency may in part account for increased SARS-CoV-2 infectivity. Since ACE2 receptor occupancy by the virus initiates infection, normal ACE2 function which plays a vital role in the cardiovascular and immune systems [109] is compromised/forfeited accounting in large part for the observed clinical sequelae. The ACE2 receptor is highly expressed in the heart and lungs [109], consistent with SARS-CoV-2 invasion of the alveolar epithelial cells, and increased secretion of ACE2 giving rise to cardiovascular and respiratory symptoms. Angiotensin-converting enzyme (ACE) and its close homologue ACE2, both belonging to the ACE family of dipeptidyl carboxydipeptidases, serve two opposing physiological functions. ACE cleaves angiotensin I to generate angiotensin II, the peptide that binds to and activates the AT1R receptor causing blood vessel constriction, thereby elevating blood pressure. Conversely, ACE2 inactivates angiotensin II while generating angiotensin 1–7, a heptapeptide having potent vasodilator function via activation of the Mas receptor [110], thus serving as a negative regulator of the renin-angiotensin system [111].

**Figure 3:**
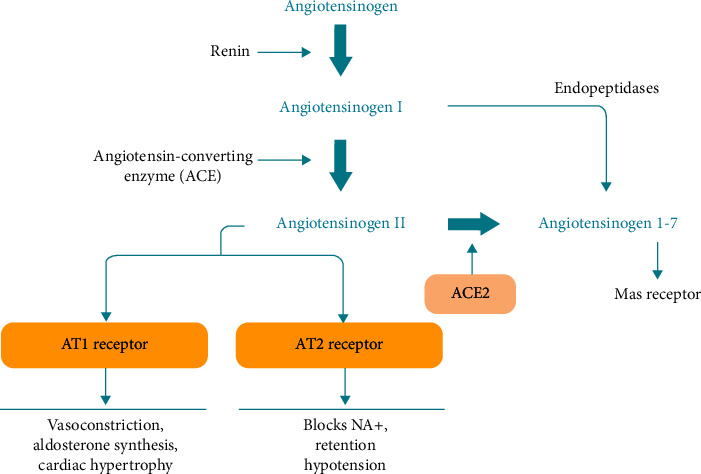
ACE signaling pathway and ACE-mediated physiological responses. Taken from Santos, R. A. S., Ferreira, A. J., Verano-Braga, T., et al., J. Endocrinol. 216, R1-R17, 2013.

Shown in Figure 3 is a schematic of the ACE signaling pathway and ACE-mediated physiological responses. The binding of SARS-CoV-2, as well as administration of SARS-CoV S protein, leads to ACE2 downregulation [111, 112] which results in increased levels of angiotensin II due to (1) ACE enzyme cleavage of angiotensin I to angiotensin II and (2) less ACE2 available for conversion of angiotensin II to the vasodilator heptapeptide angiotensin 1–7 [113]. This in turn contributes to lung injury, as angiotensin II stimulated AT1R (angiotensin II type 1a receptor) gives rise to increased pulmonary vascular permeability, thereby mediating increase lung pathology [112, 114]. Administration of ACE2 and AT1R has been shown to protect mice from severe acute lung injury, whereas ACE and its cleavage product angiotensin II promote disease pathology inducing lung edemas and impairment of lung function [114].

## Figures and Tables

**Table 1:** CoV S protein recognition elements.

Host receptor	Substrate-ligand	CoV S ligand (RBD)^a^
CD13^b^ [8]	Terminal amines	S_1_-CTD^c^
DPP4^d^ [9]	Terminal amines	S_1_-CTD
CEACAM1^e^ [10]	Cell-cell adhesion lectin	S_1_-NTD^f^
Neuraminic acid [11]	Cell, viral lectins	S_1_-NTD
ACE2^g^ [12]	Angiotensin	S_1_-CTD
APN^h^ [13]	Terminal amines	S_1_-CTD

^a^S-protein receptor-binding domain, ^b^Metalloprotease, ^c^S-protein C-terminal domain, ^d^Dipeptidase, ^e^Carcinoembryonic antigen-related cell adhesion molecule 1, ^f^S-protein N-terminal domain, ^g^Angiotensin-converting enzyme 2, ^h^Aminopeptidase N,  = reference.

**Table 2:** Overview-SARS-CoV-2 S protein processing.

Infection stage	Location	Protease
Viral attachment	Cell surface	Furin/TMPRRSS2
Viral endocytosis	Endosome/lysosome	Cathepsins L/B and furin
Viral packaging	Virus producing cell	Furin
Viral release	Extracellular space	Elastase
